# Hydrogel-Based Vascularized Organ Tissue Engineering: A Systematized Review on Abdominal Organs

**DOI:** 10.3390/gels10100653

**Published:** 2024-10-12

**Authors:** Filippos F. Karageorgos, Maria Alexiou, Georgios Tsoulfas, Aleck H. Alexopoulos

**Affiliations:** 1 Department of Transplantation Surgery, Center for Research and Innovation in Solid Organ Transplantation, Aristotle University of Thessaloniki School of Medicine, 54642 Thessaloniki, Greece; filipposk@auth.gr (F.F.K.); malex@auth.gr (M.A.); 2 Chemical Process & Energy Resources Institute, 6th Km Harilaou-Thermi Rd., P.O. Box 60361, 57001 Thessaloniki, Greece

**Keywords:** artificial organ, vascularized organ tissue engineering, hydrogels, bioprinting, artificial liver, artificial kidney, artificial pancreas, artificial intestine, abdominal organs, tissue engineering

## Abstract

Background: Biomedical engineering, especially tissue engineering, is trying to provide an alternative solution to generate functional organs/tissues for use in various applications. These include beyond the final goal of transplantation, disease modeling and drug discovery as well. The aim of this study is to comprehensively review the existing literature on hydrogel-based vascularized organ (i.e., liver, pancreas, kidneys, intestine, stomach and spleen) tissue engineering of the abdominal organs. Methods: A comprehensive literature search was conducted on the Scopus database (latest search 1 September 2024). The research studies including hydrogel-based vascularized organ tissue engineering in the organs examined here were eligible for the review. Results: Herein, 18 studies were included. Specifically, 10 studies included the liver or hepatic tissue, 5 studies included the pancreas or pancreatic islet tissue, 3 studies included the kidney or renal tissue, 1 study included the intestine or intestinal or bowel tissue, 1 study included the stomach or gastric tissue, and 0 studies included spleen tissue. Conclusion: Hydrogels are biocompatible materials with ideal characteristics for use as scaffolds. Even though organ tissue engineering is a rapidly growing field, there are still many obstacles to overcome to create a fully functional and long-lasting organ.

## 1. Introduction

### 1.1. Health Necessity of Manufacturing Organs and Organ Shortage

Transplantation consists of the golden standard in many end-stage diseases. Currently, the growing population and the modern way of living create a recipe for an increasing demand for organ transplantation, including the liver, kidneys and heart. Every day, physicians diagnose people with end-stage diseases (e.g., end-stage kidney disease and end-stage liver disease) who need to undergo a transplantation surgery. The modern way of living in combination with the growing population and the growing life expectancy create an increasing gap between the supply and demand of transplantable organs [1,2]. Hopefully, it is believed that the field of tissue engineering will be able to manufacture artificial organs suitable for transplantation.

### 1.2. Hydrogels in Tissue Engineering

Hydrogels are biocompatible materials constituting of crosslinked polymeric chains. They have the ability to absorb water and not dissolve in it due to their crosslinks. They have adjustable physical and chemical properties and are considered as one of the most suitable materials for tissue engineering applications and in general biomedical applications [3,4,5]. The characteristics of hydrogels (mesh size, molecular weight between crosslinks, polymer volume fraction in the swollen state and elastic and storage moduli) can be tuned in order to create a suitable microenvironment for the desired application. For example, the mesh size (*ξ*) and the molecular weight between crosslinks (*M_c_*) can be tuned in order to create the desired drug diffusion coefficient or the desired microenvironment for the enclosed cells so as to mimic the extracellular matrix (ECM) in terms of stiffness and pores. Extra advantages arising from the use of hydrogels are the possibility of containing cell-binding sites such as ECM proteins, a high water content and the efficient exchange of metabolites.

Nevertheless, an important problem that limits the use of hydrogels regarding liver tissue engineering applications is the difficulty in integrating different types of tissues, cell structures and cell types in the same scaffold. An additional obstacle is the process of cell migration and proliferation. That is to say that the materials (i.e., hydrogels) cannot fully mimic the pore size distribution of the natural ECM. Moreover, the integrity (e.g., stiffness) of the material, as expressed by the elastic modulus, can vary and mismatch with that of the natural ECM, which can create mechanical shear stress at the interface. Finally, hydrogel–cell adhesion can also be an issue inside a hydrogel. It is something that has to be tuned separately from other parameters via proper material selection (i.e., monomer and branching groups) of the polymer chains.

In organs such as the liver, the ECM consists of a complex mixture of molecules, each of which contributes to the final nature of the material. Therefore, the hydrogels consisting of one type of chain or having unmodified chains with various enhancing molecules may have limitations in their similarity to the ECM and use.

Overall, hydrogels consist of a class of materials with great potential in their usage in liver or kidney tissue engineering (and in other applications of tissue engineering), but rational design with the optimization of the manufactured material with the necessary utilization of mathematical models [5,6,7] has to be included in the process.

### 1.3. Scope of This Review

The scope of this systematized review [8,9] is to find the cases of hydrogel-based vascularized organ (i.e., liver, pancreas, kidneys, intestine, stomach and spleen) tissue engineering, present them and provide a critical discussion regarding the steps that are necessary for creating fully functional and vascularized artificial organs/tissues.

## 2. Materials and Methods

### 2.1. Study Design

This study was conducted partially based on the PRISMA checklist [10]. Only research papers investigating hydrogel-based vascularized organ tissue engineering were included in the study. The organs included were the liver, pancreas, kidneys, intestine, stomach and spleen. The exclusion criteria were as follows: (i) review articles; (ii) data papers; (iii) overviews; (iv) articles creating a vascularized hydrogel but not creating functional tissue/organs from those studied here (e.g., creation of cardiac tissue); (v) articles that created tissue but did not create vascular networks or somehow induce the production of a vascular network and (vi) articles creating tissue from an organ not studied here. No non-English works were found. No search filter restrictions were applied, such as a publication date. Note that the term “functional tissue/organ” in this study includes applications using endothelial cells and one type of cell, either parenchymal cells from the tissue/organ created or cells to differentiate into parenchymal cells from the tissue/organ created that can potentially form a molecule in the organ/tissue (e.g., albumin creation in liver) or that potentially perform a function that occurs in the organ or tissue (e.g., reducing sugar levels in the pancreas).

### 2.2. Search Strategy

For the search of results, one database (i.e., Scopus database) was utilized. The search term “TITLE-ABS-KEY ((vascularized) AND (liver OR hepatic OR pancreas OR pancreatic OR islet OR renal OR kidney OR nephron OR intestine OR intestinal OR bowel OR spleen OR stomach OR gastric) AND (tissue AND engineering) AND (hydrogel))” was used. FFK conducted the literature search and the selection process of the articles.

### 2.3. Data Extraction

FFK extracted the relevant data, including the year of publication, the authors, the type of study (i.e., in vitro study or in vivo study), whether the study included simulation tools or mathematical models, the organs/tissues created, the method used for the production of the matrix that included hydrogel(s) and cells, a basic outcome summary and the material used for the hydrogel.

### 2.4. Data Presentation

A series of tables (i.e., Table 1 and Table 2) present the characteristics of the included studies.

## 3. Results of Recent Advances

### 3.1. Study Selection

**Figure 1:**
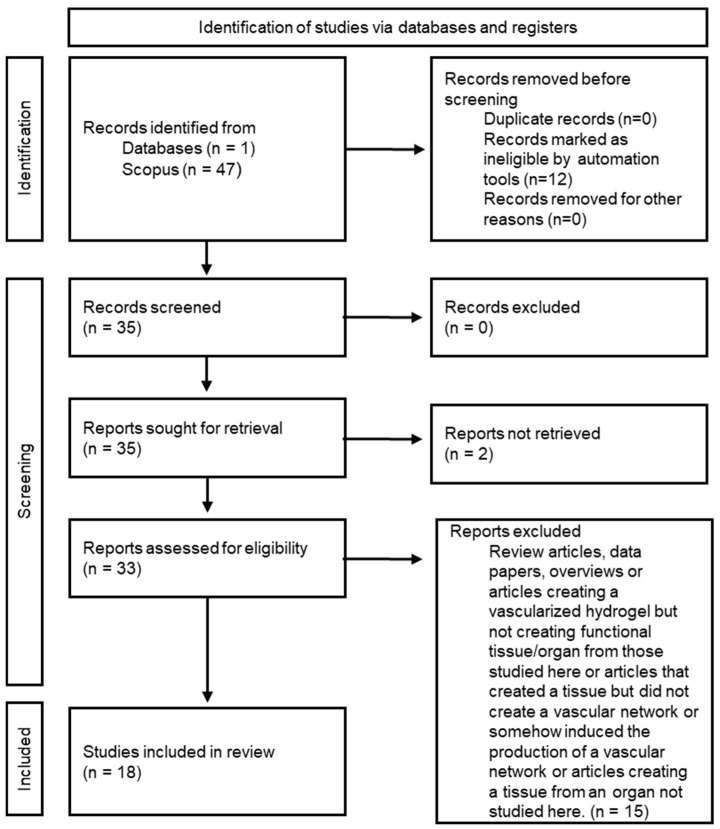
PRISMA flowchart of article selection [10].

From the literature search described in the Section 2, a total of 47 articles, books and book chapters have been found. From this number, 35 articles were considered eligible for the study (note that in this step, the articles were eligible according to Scopus, and no review or data papers were included), and full-text examination and data extraction was performed. Finally, after removing non-eligible studies (according to the exclusion criteria mentioned previously (Section 2)) and not being able to find 2 papers, 18 studies were considered for the review [11,12,13,14,15,16,17,18,19,20,21,22,23,24,25,26,27,28] (Figure 1).

### 3.2. Study Characteristics

All the studies that were included in the review were published from 2010 to 2024. The studies varied, including different organs/tissues for creation in each or more than one organ/tissue. The studies also varied, since some included in vitro results, some were in vivo, and some included both types (i.e., in vitro and in vivo). A wide range of production methods for the hydrogels was used, including a single syringe, a double syringe, 3D bioprinting, light-induced crosslinking, temperature-induced crosslinking and more (for details see Table 1). Regarding the materials used, collagen, alginate and fibrin are mostly utilized (for more details, see Table 1). The studies’ results are not comparable, since the studies included different materials, cell lines and production methods. Even the culture periods in the in vitro and in vivo studies differed from a few days to 14 weeks [22]. From the eighteen studies, there was one particular study [15] where the authors wanted to see how a system of organs/tissues/organoids could function in an in vitro environment. The authors included a triculture of small intestinal organoids, stomach organoids and induced hepatic tissues co-cultured with HUVECs [15]. The in vitro environment consisted of intestinal media, gastric media and hepatic induction media [15]. Regarding each organ/tissue studied here, 10 results [11,12,13,14,15,16,17,18,19,20] included liver tissue, 5 results [21,22,23,24,25] included pancreatic islet tissue, 3 results included kidney tissue [18,26,27], 1 result included intestinal tissue [28], and 1 result included gastric tissue [16]. Unfortunately, there were no studies including hydrogel-based organ tissue engineering for the spleen. In addition, some studies were for healthy tissues and some for cancerous tissues. The time of testing for each in vivo study was from a few days (i.e., most of the studies) up to 14 weeks (for study [22]). Also, two studies for liver tissue/organ [13,15] included decellularized tissue for their hydrogels.

## 4. Discussion

### 4.1. Summary of the Results

From the results of the study, it can be concluded that there is a limited number of applications involving hydrogel-based vascularized tissue/organ creation with both endothelial and parenchymal cells. This limited number of findings might be attributed to the use of only the Scopus database. Additionally, the majority of the tissues constructed here are mainly for the liver (10/18 ≅ 0.556), pancreas (5/18 ≅ 0.278) and kidney (3/18 ≅ 0.167). In addition, equal work has been found for stomach and intestinal hydrogel-based tissue engineering (1/18 ≅ 0.056), and finally, nothing was found on the creation of a hydrogel-based vascularized spleen (0/18 = 0). The latter could be attributed to the fact that even though the spleen is an important part of the human immune system, one can survive without this organ. It is also true that one can live without their pancreas or having diabetes mellitus type I but not without side effects. Specifically. in the first case, there will be problems in regulating blood sugar and in nutrient absorption from food. In the latter case, only blood sugar-regulating problems appear.

From the current applications, it is evident that there are no applications that have been tested for a period of 6 months and more. All these presented applications are studied mostly in the range of days to weeks. If these applications want to proceed in human therapeutic applications, the long-term stability is a crucial issue to pass or fail one application. This information has been added to the revised manuscript. This is a testing period limitation of all these applications, since eventually, they cannot be trusted for long-term usage and potentially for tissue/organ substitution. One of the negative outcomes found from this systematized review is that there is not a universal hydrogel matrix that can be used in hydrogel-based tissue engineering applications. At least the authors use different matrices most of the time, meaning that there is still room for improvement in the material selection. A noteworthy observation is that two studies are using hydrogels made from decellularized tissue from the tissue they are trying to produce [13,15]. Note that the decellularized tissue includes all the ECM elements (e.g., GAG) of the source tissue. This makes the decellularized hydrogel closer to the natural ECM than other hydrogel constructs. Moreover, is worthy to mention that most of the applications in Table 1 are using naturally derived materials (i.e., pre-polymers) for the hydrogels, such as gelatin and fibrin.

One of the positive facts is that the studies seemed to increase in number yearly as we reached the current year. Additionally, it can be observed that more complicated, more advanced and more reliable methods are being created and used in the production of hydrogel-based tissue/organ engineering. These methods include the transition from a single syringe to make a hydrogel to a double syringe to a 3D bioprinter to organs in a chip to robot-automated production of hydrogel-based vascularized organs for tissue engineering. In fact, this can make the results better, more sophisticated and more reliable. Also, the constructs can be more detailed and advanced, allowing them to have more possibilities to perform the job they were designed to.

Another thing that we observed is that there are mainly two strategies to make a vascularized network. The first utilizes a gel patch that is implanted in an organism, which later leads to the growth of a vascularized network. The other strategy includes the creation of pre-vascularized networks that can be implanted or can be used as a drug testing model. Judging by the current findings, it is more possible to design and manufacture drug testing devices than to construct a tissue/organ substitute.

There are efforts to create tissue/organ-like structures capable of being used as a drug testing model [15].

### 4.2. State-of-the-Art Applications

Summarizing for each organ, the state of the art could not be provided definitively, as the technologies under investigation are following different approaches with different obstacles and uncertainties and are at different stages of development. For instance, the state of the art could be a technology used in vivo, which provides the closest to human use, but in the case of drug toxicity testing, the organ-on-a-chip applications could be considered the state of the art, since without risking any life, a toxicity test could be conducted for numerous drugs. So, the final state-of-the-art technology might not be a single one, but the manufacturing method and the potential use should be taken into account as significant aspects of the technologies under consideration.

Taking this into consideration, for the hepatic organ/tissue, the applications from references [18,19,20] could be considered the state of the art, since reference [18] includes a subtractive manufacturing technique to create the matrix, reference [19] describes a microfluid chip that combines 3D bioprinting and the use of robotic handling, and reference [20] also uses a bioprinting method. For the pancreatic tissue/organ, the state-of-the art title could be given to the applications of reference [22], since their construct was applied to their in vivo experiments for up to 14 weeks, utilizing a two-component synthetic PEG hydrogel. For the kidney organ/tissue, reference [27] could be considered as state of the art, since the authors used their application in in situ renal injury repair, improving the injured renal microenvironment. For the intestinal tissue-related applications, [28] could be considered state of the art, since the authors created an organoid on a chip with multiple potential uses. Finally, for stomach or gastric organ/tissue, the application from reference [16] can be considered state of the art.

Finally, due to the number of applications, it is difficult to find a trend over the years on the production methods and the materials used.

### 4.3. Comparison of Findings from the Literature

From the research, there were no similar systematized or systematic reviews or reviews for hydrogel-based organ/tissue engineering with an emphasis on hydrogel materials and applications in the abdominal organs that the current review focuses on. There were, however, many interesting non-systematized and non-systematic reviews that include tissue engineering in vascularized composite allotransplantation from Ren et al. [29] or include vascularized organ-on-a-chip systems from Fritschen and Blaeser [30]. Additional interesting reviews include the use of biomaterials for vasculature engineering to construct liver tissue from Lv et al. [31] or include various biomaterials to promote vascularization in organ tissue engineering from Zhao et al. [32]. Finally, a really interesting and detailed review article has been published by Rojek et al. [33] on microfluidic formulation of topological hydrogels for microtissue engineering. This article focuses on microtissue engineering applications, and specifically, it reviews the available microfluidic fabrication methods and critically discusses the available tissue-specific applications.

### 4.4. Limitations of the Review

Various limitations can be attributed to this systematized review [8,9]. First of all, the article database usage was limited, meaning that more databases should have been used in the initial search. The snowballing technique could also be used. Moreover, the screening and analysis of articles could be conducted by a second person, thus rendering this review a systematic review. Finally, more organs could be included (e.g., heart, lungs, reproductive organs, etc.).

## 5. Conclusions

In summary, the field of vascularized organ tissue engineering shows a potential solution and research direction to follow regarding artificial organs. Even today, the results, although important, are still not enough to talk about potential organ replacement. The recent applications show the capability of achieving complex tissue creation using various methods, including double syringes for producing the hydrogel matrix, 3D printing with hydrogel-based materials, robot-automated usage for increased precision and speed, hydrogels including a decellularized ECM, microfluidics, organ on a chip and more. Some of the works include pre-vascularized tissues that can be implanted; others include tissues in which the vascularized network will be developed after implantation. In fact, the applications found here, even the in vivo studies, have not been tested for long-enough periods to see the longevity of these interventions for 6 to 12 months. A lot of work has to be conducted to mimic the complexity of human organs that include various tissue cells in a vascularized final matrix.

## 6. Future Directions

For future directions, there are many things that could be described. The major requirement in the current state is to increase the number of applications/research efforts and combine current technologies. Secondly, the research groups should proceed with technologies/applications that can work in vivo to replicate an organ/tissue and be efficient and functional for a prolonged period of time of 6 month to a year. Thirdly, the research groups should address the problem on its basis, meaning that it is not necessary to make an organ that has the same characteristics and complexity and shape of the impaired organ/tissue but rather to realize the job adequately. Fourthly, the tissues are not just endothelial cells and parenchymal cells. For example, the liver includes a series of cells such as hepatocytes, Kupffer cells and liver sinusoidal endothelial cells, among others. Finally, with the current technological advancements presented here, there should be more involvement of computational simulations and mathematical models to reduce the development time, total effort and financial resources for the in vitro pre-clinical phase. In addition, these methods can also reduce the sacrificed animals for the in vivo pre-clinical phase.

Finally, the researchers have to focus on a major aspect of the creation of a hydrogel-based artificial organ/tissue. This is bioprinting and the ethical dilemmas that arise with it. Even although bioprinting can avoid the ethical dilemmas associated with clinical organ transplantation and/or xenotransplantation, it has unique challenges, which are necessary to address. It is of high importance to define the role of bioprinting organs/tissues and to clarify the limitations and the ethical perspectives. The process of bioprinting is in its infancy, which means that there is a plethora of parameters that are mandatory to be considered [34]. We provide two interesting works for the readers to deepen their knowledge regarding ethics and bioprinting: [35,36].

## Figures and Tables

**Table 1:** Extracted relevant data, including the year of publication, authors, the type of study, whether simulation tools or mathematical models were mentioned to be used, the organs/tissues created, the method used for the hydrogel preparation/gelation and the material used for the hydrogel, from the selected publications.

No.	Year Published *	Authors	Type of Study	Used Simulation Tools	Organs/ Tissues Created	Method Used for Hydrogel Preparation/Gelation	Material Used for Hydrogel	In Vivo Experiment Duration
Liver/hepatic tissue-related applications								
1	2010	Zhao Y. et al. [11]	In vivo (rats)	−	Hepatic tissue	Gel was prepared in a syringe	Collagen	−
2	2013	Leong M.F. [12]	In vitro/in vivo (mice)	−	Hepatic tissue	After polyionic droplets were arranged on a template, individual fibers were drawn in parallel and assembled into higher-order structures. Cells were encapsulated in the fiber.	Alginate	Animals were sacrificed on day 14
3	2018	Agarwal T. et al. [13]	In vitro	−	Liver tissue	After the pH of the solution was adjusted, the pre-gel solutions were prepared on ice to prevent the gelation of the matrix. Thereafter, it was briefly centrifuged, added to the culture dish and incubated at 37 °C for 20 min to facilitate gelation. Cells were entrapped into hydrogels post-neutralization	Caprine liver extracellular matrix (CLECM) and collagen	−
4	2018	Zhang B. et al. [14]	In vitro	−	Hepatic tissue	Collagen–Matrigel hydrogel mixture was prepared. Then, the cells were suspended in the mixture. The bioreactor was placed in an incubator with a built-in humidified chamber for 30 min to achieve the gelation of collagen–Matrigel.	Collagen–Matrigel	−
5	2018	Jin Y. et al. [15]	In vitro	+	Hepatic tissue and multiorgan model (3D liver organoids; 3D gastric and small intestinal organoids)	The decellularized liver extracellular matrix (LEM) was used to form a 3D hydrogel by inducing the self-assembly of extracellular matrix (ECM) components by adjusting the temperature. The cells were resuspended in the LEM pre-gel solution and incubated at 37 °C for 30 min to induce gelation. In addition, gastric organoids and intestinal organoids were encapsulated in Matrigel.	Liver extracellular matrix (LEM) as main ingredient for liver, with Matrigel for gastric and intestinal tissue	−
6	2019	Carpenter R. et al. [16]	In vivo (mice)	−	Liver tissue	Interconnected porous hydrogel scaffolds were created. For their fabrication, glass beads were utilized, and the precursor solution was composed of an acrylamide monomer, bisacrylamide crosslinker, N,N,N′,N′-tetramethylethylenediamine accelerator and 2-hydroxy-2-methylpropiophenone photoinitiator in nitrogen-purged DI water. The precursor solution was immediately polymerized under a UV light source for 15 min. Glass beads were selectively dissolved. Stromal cells were seeded in the scaffolds. And tissue pieces were inserted in a hole in the scaffold or on top of the scaffold.	Acrylamide monomer was the main ingredient of the hydrogel	2 weeks for liver tissue/organ
7	2020	Cui J. et al. [17]	In vitro	+	Liver lobule-like tissue	Each of the two pre-polymer solutions (i.e., PEGDA and GelMA) were fully mixed with cell-laden solutions. Then, photo-crosslinking took place to form the hydrogels.	Poly(ethylene glycol) diacrylate (PEGDA) and gelatin methacrylate (GelMA)	−
8	2022	Rajasekar S. et al. [18]	In vitro	−	Vascularized liver tissue	The authors developed a subtractive manufacturing technique. Using this technique, a 2D surface can be patterned using a flexible sacrificial material that changes its shape and swells when it is exposed to an aqueous hydrogel. It then subsequently degrades to create perfusable networks in a natural hydrogel matrix that can be populated with cells.	Alginate, collagen, Matrigel and fibrin	−
9	2024	Fritschen A. et al. [19]	In vitro	−	Liver carcinoma	Microfluidic chip in combination with the automated 3D-bioprint process and robotic handling.	Agarose and fibrin	−
10	2024	Jiang Z. et al. [20]	In vitro/in vivo (mice)	−	Liver	Cells from a C166 suspension or from a hepatocyte suspension were mixed with GelMA solution and LAP solution at 37 °C to bioprint cell-laden hepato-spheroid-encapsulated artificial livers with veins. Syringes loaded with inks were stored at 4 °C for 20 min until complete physical gelation. Then, the syringes were mounted into the extruders on the 3D printer with a temperature at 20 °C. Finally, they were UV irradiated for 30 s to crosslink the printed constructs.	Gelatin methacrylate (GelMA)	2 weeks after transplantation
Pancreatic tissue-related applications								
11	2018	Jung et al. [21]	In vivo (mice)	−	Pancreatic cancer tissue	Disc-shaped fibrin samples were fabricated by casting a mixture of fibrinogen and thrombin (specifically thrombin and aprotinin) solutions in PDMS wells. During mixing, cell culture medium containing cells was added to the fibrinogen solution.	Fibrin	Up to 8 weeks
12	2018	Weaver J.D. et al. [22]	In vivo (rats)	+	Islet transplantation with vascularization	A hydrogel core crosslinked with a non-degradable PEG dithiol and a vasculogenic outer layer crosslinked with a peptide that is susceptible to proteases to facilitate degradation comprise a two-component synthetic PEG hydrogel macro-device system.	Poly(ethylene glycol) (PEG) as the major ingredient	4 weeks after transplantation for glucose monitoring; up to 14 weeks after transplantation for stability
13	2022	Hsu Y.-J. et al. [23]	In vitro/in vivo (mice)	+	Islet transplantation	Pre-polymer GelPhMA solutions were gently mixed with mesenchymal stem cells alone or a mixture of mesenchymal stem cells and human umbilical endothelial cells. Then, photo-crosslinking with UV light occurred.	Gelatin–phenolic hydroxyl-methacrylic anhydride (GelPhMA)	Up to 1 week
14	2022	Kinney S.M. et al. [24]	In vivo (mice were recipients; donors were rats)	−	Subcutaneous islet transplantation	The injectable hydrogels were prepared as follows: Precursor solutions were quickly drawn up in a syringe capped with an 18-gauge needle and were then used to quickly draw islet-equivalent units into the needle. The mixture of islets and hydrogel was injected into the mice approximately 30 s prior to the gelation time of the hydrogels.	Methacrylic acid-polyethylene glycol (MAA-PEG)	Up to 70 days
15	2022	Perugini et al. [25]	In vitro	−	Vascularized pancreatic β-cell islets	In a gelatin-containing medium, β-cells and endothelial cells were suspended. After putting the cell suspension in sterile tubes, it was then left at 37 °C for 20 min on a tissue culture rotator. Then, the cells, after a 2 h incubation, were treated with fetal bovine serum and incubated for another 24 and 48 h under continuous, gentle agitation at 37 °C, 5% CO_2_ and 95% humidity, which was sufficient to improve media flow around the formed 3D constructs.	Gelatin	−
Renal tissue-related applications								
16	2019	Huling J. et al. [26]	In vitro (rats)	−	Vascularized functional renal tissues	The MS1-coated scaffolds were embedded in collagen type 1 hydrogel in 48-well plates. Human renal cells were mixed in the collagen hydrogel before they were neutralized with NaOH.	Polycaprolactone (PCL) and collagen were used in the application	−
17	2022	Rajasekar S. et al. [18]	In vitro	−	Vascularized kidney proximal tubules	The authors developed a subtractive manufacturing technique. Using this technique, a 2D surface can be patterned using a flexible sacrificial material that changes its shape and swells when it is exposed to an aqueous hydrogel. It then subsequently degrades to create perfusable networks in a natural hydrogel matrix that can be populated with cells.	Alginate, collagen, Matrigel and fibrin	−
18	2023	Zhang Y. et al. [27]	In vitro/in vivo (rats)	−	Renal tissue	Photo-crosslinking (UV light) for hydrogel preparation and then cell seeding in vitro and photo-crosslinking (UV light) for hydrogel preparation in vivo.	Gelatin methacrylate (GelMA)	Up to 12 weeks
Intestinal tissue-related applications								
19	2024	Orge I.D. et al. [28]	In vitro	−	Vascularized intestinal organoids	A fibrinogen solution mixed with aprotinin was used. For hydrogel embedding, intestinal organoids combined with vascular units, these were added to the fibrinogen solution and then mixed with thrombin and allowed to crosslink for 30 min at 37 °C.	Fibrin-based hydrogels were mainly used	−
Stomach tissue-related applications								
20	2019	Carpenter R. et al. [16]	In vivo (mice)	−	Gastric cancer tissue	Interconnected porous hydrogel scaffolds were created. For their fabrication, glass beads were utilized, and the precursor solution was composed of an acrylamide monomer, bisacrylamide crosslinker, N,N,N′,N′-tetramethylethylenediamine accelerator and 2-hydroxy-2-methylpropiophenone photoinitiator in nitrogen-purged DI water. The precursor solution was immediately polymerized under a UV light source for 15 min. Glass beads were selectively dissolved. Stromal cells were seeded in the scaffolds. And tissue pieces were inserted in a hole in the scaffold or on top of the scaffold.	Acrylamide monomer was the main ingredient of the hydrogel	6 weeks for gastric tissue/organ

* The year has been taken from the Scopus results list.”-“ means no and “+” means yes in the fifth column.

**Table 2:** Basic outcome summary of the selected studies.

No.	Year Published *	Authors	Outcome Summary
Liver/hepatic tissue-related applications			
1	2010	Zhao Y. et al. [11]	Type I collagen hydrogel was used as a matrix for the growth and differentiation of hepatocytes to create engineered hepatic units to reconstitute 3D, vascularized hepatic tissue in vivo. The hepatocytes were transplanted in vivo in small hepatic units. Large hepatic tissue (more than 0.5 cm thick) containing blood vessels could be engineered in vivo by merging small hepatic units
2	2013	Leong M.F. [12]	Patterned constructs of endothelial cells with anastomosing hepatocytes with the host vasculature in a mouse model, leading to vascularized tissue.
3	2018	Agarwal T. et al. [13]	The co-culture of HepG2 cells and endothelial cells in decellularized caprine liver extracellular matrix-derived hydrogel showed functionality and differentiation for both cell types. It also showed that these hydrogels supported the development of the microvasculature in vitro. This renders it a suitable candidate for development of a pre-vascularized liver tissue construct
4	2018	Zhang B. et al. [14]	A detailed protocol is provided for fabricating the AngioChip scaffold, populating it with endothelial cells and parenchymal tissues. The functionality of AngioChip-vascularized hepatic tissue was demonstrated.
5	2018	Jin Y. et al. [15]	Vascularized liver organoid-like tissue constructs were generated via the 3D co-culture of induced hepatic (iHep) cells and endothelial cells in a 3D decellularized liver extracellular matrix (LEM) hydrogel reconstituted in a microfluidic device. The resulting 3D vascularized liver organoids showed enhanced drug responses, metabolic activity, biosynthetic activity and hepatic functionality under this physiologically relevant culture microenvironment. In addition, the 3D liver organoids were also placed in a tripartite culture along with 3D gastric and small intestinal organoids. The feasibility of using the iHep-based 3D liver organoid is proven. It can be used as a high-throughput drug screening platform, and it can also be used in a multiorgan model consisting of multiple internal organoids.
6	2019	Carpenter R. et al. [16]	It was found that the human bone marrow stromal cell scaffold improved the preservation of intrinsic hepatic sinusoids and assisted in reaching stably reconnected vasculature compared to scaffold-free and blank-scaffold results. Blank-scaffold and human bone marrow stromal cell scaffold-transplanted hepatic tissues retained morphological features present in the native liver (e.g., portal veins). The characterization of albumin secretion via immunohistochemistry revealed the maintenance of liver function in all transplanted tissues.
7	2020	Cui J. et al. [17]	A ten-layered liver lobule-like construct with an embedded lumen, containing an inner radial-like poly(ethylene glycol) diacrylate structure with hepatocytes and outer hexagonal gelatin methacrylate structure with endothelial cells, was assembled. The 3D liver lobule-like constructs demonstrated high cell activity throughout long-term co-culture. They also maintained tissue-like morphology and the vascular lumen. The 3D liver lobule-like constructs allowed for perfusion culture through its lumen, which promoted albumin secretion of the embedded cells.
8	2022	Rajasekar S. et al. [18]	The developed technique was applied to fabricate organ-specific vascular networks and then further scaled to a 24-well plate format to make a large vascular network, vascularized liver tissues and for integration with ultrasound imaging.
9	2024	Fritschen A. et al. [19]	The functionality of the developed microfluidic chip in combination with the automated 3D-bioprint process and robotic handling is demonstrated. A liver carcinoma model based on HepG2 cells is presented, and a stable microvascular network is developed around the islets of the HepG2 cells. The HepG2 cells proliferate strongly and form spheroidal agglomerates
10	2024	Jiang Z. et al. [20]	By combining with compatible bioink a bulk gel support medium called omnidirectional printing embedded network, the researchers were able to generate hepato-spheroid-encapsulated artificial liver constructs that maintain liver function in vitro and promote neovascularization in vivo.
Pancreatic tissue-related applications			
11	2018	Jung et al. [21]	Endothelial cells and pancreatic carcinoma cells were encapsulated in fibrin gel and subcutaneously implanted into nude mice. It was shown that 3D cancer xenograft tissue formation may be achieved in vivo by using fibrin hydrogel as a scaffold and endothelial cells to support vasculogenesis.
12	2018	Weaver J.D. et al. [22]	A synthetic, non-degradable hydrogel microdevice designed to encapsulate and deliver islets to the omentum was presented. The vasculogenic and degradable hydrogel layer that was applied to the macro-device’s exterior interface improved the vascular density in the rat omentum transplant site in vivo. This resulted in improved encapsulated islet viability in a syngeneic diabetic rat model.
13	2022	Hsu Y.-J. et al. [23]	Using an animal model that is clinically relevant, a cell-based approach for rapidly generating large, functional vasculature was developed and shown to possess advantages over existing techniques. The hydrogel structures’ geometry was optimized by the utilization of diffusion-based computational simulations. Furthermore, transplanted islets were rapidly integrated subcutaneously in this developed functional vascular bed, which significantly improved islet viability and insulin secretion.
14	2022	Kinney S.M. et al. [24]	It was demonstrated that islets engraft in the subcutaneous space when injected in an inherently vascularizing, degradable methacrylic acid–polyethylene glycol (MAA-PEG) hydrogel. Note that no vascularizing cells or growth factors were required. In diabetic mice, the injection of 600 rodent islet equivalents in MAA-PEG hydrogels was sufficient to reverse diabetes for 70 days. Additionally, a PEG gel without MAA had no benefit. Over the course of a week, the MAA-PEG hydrogel scaffolds degraded and were replaced by a host-derived, vascularized, innervated matrix that supported subcutaneous islets. Islets delivered in degradable hydrogels were incorporated into a host-derived, vascularized, collagen-rich subcutaneous matrix by the 21st day.
15	2022	Perugini V. et al. [25]	The formation of bioengineered pancreatic islets through cell anchoring to a gelatin-based biomaterial, PhenoDrive-Y, which can mimic the basement membrane of tissues, was demonstrated. This contrasts with the majority of studies published till this date, where pancreatic islets of pancreatic β-cells are encapsulated in hydrogels. Gelatin was used as control. The cultures with PhenoDrive-Y exhibited a greater degree of organization in tissue-like structures, more pronounced endothelial sprouting and stronger expression of characteristic/typical cell markers in comparison to gelatin. In addition, the two constructs were incubated in normo-glycemic conditions and hyperglycemic conditions.
Renal tissue-related applications			
16	2019	Huling J.et al. [26]	In a rat renal cortical defect model, a biomimetic collagen vascular scaffold achieved by means of a vascular corrosion casting technique can improve vascularization within the collagen hydrogel implant. The created scaffolds were coated with endothelial cells to pre-vascularize them. They were then integrated into three-dimensional renal constructs and implanted in the renal cortexes of nude rats, either with or without human renal cells. The collagen-based vascular scaffold that was implanted was easily identified and integrated into native kidney tissue. When combined with human renal cells, the biomimetic collagen vascular scaffolds coated with endothelial cells can improve vascularization and assist the formation of renal tubules after 14 days.
17	2022	Rajasekar S. et al. [18]	The developed technique was applied to fabricate organ-specific vascular networks, vascularized kidney proximal tubules, in a customized 384-well plate.
18	2023	Zhang Y. et al. [27]	A novel, biocompatible, 3D, porous gelatin methacrylate (GelMA) hydrogel (DFO-gel) with sustained releasing of deferoxamine (DFO) and a prolonged hypoxia-mimicking environment was developed using a facile and feasible photo-crosslinking method (using UV light) for in situ renal injury repair. This hydrogel can also improve the injured renal microenvironment by alleviating oxidative and inflammatory stresses, accelerating neovascularization and promoting efficient anti-synechia
Intestinal tissue-related applications			
19	2024	Orge I.D. et al. [28]	By integrating vascularized microtissues or vascular units with intestinal organoids within the “vessel-on-chip”, it is illustrated how vascular units can act as “vascularization bridges”, extending a microvascular network throughout the hydrogel bulk and supporting the formation of a vascularized stroma around multiple organoids within a single microfluidic device.
Stomach tissue-related applications			
20	2019	Carpenter R. et al. [16]	Regarding gastric cancer, it was found that tumor morphology appeared to be conserved following transplantation in successfully engrafted tissue. In addition, scaffold-assisted transplantation did not negatively affect the tumor phenotype compared to the control. Also, immuno-histostaining results demonstrate higher mitogenic and human immune cell activities in human tumors co-implanted with biomaterials compared to that of scaffold-free controls.

* The year has been taken from the Scopus results list.

## Data Availability

Data sharing is not applicable, since no new data were generated.
